# The small phytoplasma virulence effector SAP11 contains distinct domains required for nuclear targeting and CIN-TCP binding and destabilization

**DOI:** 10.1111/nph.12721

**Published:** 2014-02-19

**Authors:** Akiko Sugio, Allyson M MacLean, Saskia A Hogenhout

**Affiliations:** 1 Cell and Developmental Biology, The John Innes CentreNorwich Research Park, Norwich, NR4 7UH, UK

**Keywords:** coiled coil, effector, host target, mollicutes, phytoplasma, plant development, TCP, virulence

## Abstract

1. Phytoplasmas are insect-transmitted bacterial phytopathogens that secrete virulence effectors and induce changes in the architecture and defense response of their plant hosts. We previously demonstrated that the small (± 10 kDa) virulence effector SAP11 of Aster Yellows phytoplasma strain Witches’ Broom (AY-WB) binds and destabilizes Arabidopsis CIN (CINCINNATA) TCP (TEOSINTE-BRANCHED, CYCLOIDEA, PROLIFERATION FACTOR 1 AND 2) transcription factors, resulting in dramatic changes in leaf morphogenesis and increased susceptibility to phytoplasma insect vectors. SAP11 contains a bipartite nuclear localization signal (NLS) that targets this effector to plant cell nuclei.
2. To further understand how SAP11 functions, we assessed the involvement of SAP11 regions in TCP binding and destabilization using a series of mutants.
3. SAP11 mutants lacking the entire N-terminal domain, including the NLS, interacted with TCPs but did not destabilize them. SAP11 mutants lacking the C-terminal domain were impaired in both binding and destabilization of TCPs. These SAP11 mutants did not alter leaf morphogenesis. A SAP11 mutant that did not accumulate in plant nuclei (SAP11ΔNLS-NES) was able to bind and destabilize TCP transcription factors, but instigated weaker changes in leaf morphogenesis than wild-type SAP11.
4. Overall the results suggest that phytoplasma effector SAP11 has a modular organization in which at least three domains are required for efficient CIN-TCP destabilization in plants.

## Introduction

Phytoplasmas are bacterial plant pathogens known for causing dramatic symptoms in their plant hosts, including changes in architecture, defense response and volatile production (Hogenhout *et al*., 2008; Mayer *et al*., 2008; Hoshi *et al*., 2009; Strauss, 2009; MacLean *et al*., 2011; Sugio *et al*., 2011a,b). These typically include witches’ brooms (excessive stem production), phyllody (floral organs that turn into indeterminate leaf-like structures) and virescence (greening of flower organs). The plant phenotypic modulations appear to benefit phytoplasma fitness through the generation of more vegetative tissues and attraction of insect vectors that disperse the phytoplasmas (Hogenhout *et al*., 2008; Mayer *et al*., 2008; Hoshi *et al*., 2009; Sugio *et al*., 2011b). Phytoplasmas cause dramatic yield losses of crops worldwide, primarily because they interfere with flower, fruit and seed production (Lee *et al*., 2000). Nonetheless, some phytoplasmas can be beneficial. For example, phytoplasmas are commonly used to induce free branching in commercial Poinsettia cultivars (*Euphorbia pulcherrima*) in which the phytoplasma-induced proliferation of shorter branches generates a more attractive plant (Lee *et al*., 1997).

Despite the fact that phytoplasmas cannot be cultured in artificial medium, much progress has been made in the characterization of virulence effector proteins that contribute to the various changes in plant phenotype. These effectors were first discovered through phytoplasma genome sequence analyses and functional genomics approaches (Bai *et al*., 2009; Hoshi *et al*., 2009). In the genome of Aster Yellows phytoplasma strain Witches’ Broom (AY-WB) more than 50 secreted AY-WB proteins (SAPs) were identified that are candidate virulence effectors (Bai *et al*., 2009). Phytoplasmas reside in the cytoplasm of sieve cells of the plant phloem. Being intracellular, phytoplasmas secrete effectors via the Sec-dependent secretion pathway in which the signal peptide is cleaved off (Kakizawa *et al*., 2004). The majority of phytoplasma effector proteins are smaller than 40 kDa and therefore, upon secretion by the phytoplasma, may unload from the sieve cells and migrate to adjacent tissues (Imlau *et al*., 1999; Bai *et al*., 2009). SAP11 was shown to predominantly target plant cell nuclei when transiently produced in *Nicotiana benthamiana* leaves and in AY-WB-infected plants (Bai *et al*., 2009), and induces stem proliferation, alterations in leaf shape and downregulation of jasmonic acid (JA) production, the latter increasing susceptibility of plants to the AY-WB leafhopper vector *Macrosteles quadrilineatus* (Sugio *et al*., 2011b). Another AY-WB effector, SAP54, induces the production of leafy indeterminate flowers that resemble the phyllody symptoms characteristic of AY-WB-infected plants (MacLean *et al*., 2011). TENGU is an effector characterized from Onion Yellows (OY) phytoplasma and induces dwarfism and witches’ brooms in plants (Hoshi *et al*., 2009; Sugawara *et al*., 2013).

So far, the plant targets of one phytoplasma effector, SAP11, have been identified. It was found that SAP11 binds and destabilizes class II CIN (CINCINNATA) TCP (TEOSINTE-BRANCHED, CYCLOIDEA, PROLIFERATION FACTOR 1 AND 2) (Sugio *et al*., 2011b). CIN-TCPs regulate various plant developmental functions, arguably the most obvious of which is leaf morphogenesis (Martin-Trillo & Cubas, 2010). Transgenic Arabidopsis plants that overexpress miR319, which is a negative regulator of five of eight CIN-TCPs, exhibit changes in leaf shape and size due to excess cell division, mainly at the leaf margins, resulting in the production of large crinkly leaves (Palatnik *et al*., 2003). This Arabidopsis leaf crinkling phenotype is more severe in plants that overexpress miR319 together with an artificial miRNA miR3TCP targeting the remaining three CIN-TCPs (Efroni *et al*., 2008). SAP11 was previously shown to bind and destabilize all eight CIN class II TCPs leading to the induction of severe leaf crinkling and downregulation of jasmonic acid (JA) that is the direct result of reduced CIN-TCP presence (Schommer *et al*., 2008; Sugio *et al*., 2011b; Danisman *et al*., 2012). Whilst SAP11 may bind class I TCPs, this effector does not appear to destabilize these TCPs (Sugio *et al*., 2011b). Class I and II TCPs have antagonistic functions in controlling plant development (Kosugi & Ohashi, 2002; Li *et al*., 2005; Martin-Trillo & Cubas, 2010; Danisman *et al*., 2012).

SAP11 is *c*. 10 kDa (90 amino acids), but nonetheless appears to encode at least three distinct activities, which are targeting of nuclei, binding of TCPs and destabilization of TCPs. Nuclear-localized virulence effectors of other bacterial pathogens are often much larger than the size exclusion limit of the nuclear pore complex, which is *c*. 60 kDa (Gorlich, 1998; Talcott & Moore, 1999) and have distinct domains involved in nuclear targeting and target binding (Rivas, 2012). To better understand how the various functions are accommodated in SAP11, we employed yeast two-hybrid analyses, agroinfiltration assays in *N. benthamiana* leaves and stable transgenic expression of SAP11 constructs in Arabidopsis to dissect the domains involved in SAP11 nuclear targeting and TCP-binding and destabilization. Surprisingly, SAP11 has a linear modular structure with different parts of the effector being involved in nuclear localization, TCP binding and TCP destabilization. We discuss our findings in the broader context of virulence effector evolution in phytoplasmas.

## Materials and Methods

### Construction of SAP11 derivatives

All of the intermediate DNA constructs were maintained in *Escherichia coli* DH5α cells. A codon-optimized version of the SAP11 sequence (Sugio *et al*., 2011b) was used to create the mutant constructs used in this study. The *SAP11ΔN* mutant was amplified by primers attB1sap11dF2 and Sap11NLS3′R, and the fragment was amplified again by attB1 and attB2 adapter primers to add complete attB sequences. The fragment was cloned into pDONR207™ by Gateway® BP Clonase® II (Invitrogen). The SAP11ΔC and SAP11ΔCΔcc mutants were PCR amplified by primer combinations of FullattBadaptSAP11F and FullattB2sap11dR1, and FullattBadaptSAP11F and FullattB2sap11dR2, respectively. The fragments were cloned into pDONR207 by BP Clonase® II. The *SAP11ΔNLS* mutant was created by PCR amplification of plasmid pDONR207-SAP11 using primers SAP11optNLS3QCF and SAP11optNLS3QCR and digestion of the template plasmid by *Dpn*I restriction enzyme. NES or NESKO sequences were attached to *SAP11* or *SAP11ΔNLS* mutant by PCR amplifying the sequences using Sap11NLS5′F and SAP11NESattB2 (for NES) or SAP11NESKOattB2 (for NESKO). The PCR products were amplified using attB1 and attB2 adapter primers and cloned into pDONR207. All the clones were sequenced to verify the sequence of the inserts. Primer sequences are shown in Supporting Information Table S1. The genes in pDONR207 were then cloned into the Gateway destination vectors.

### Generation and analyses of transgenic Arabidopsis lines

pB7WG2 (Karimi *et al*., 2002) with SAP11 derivatives were transformed into *Agrobacterium tumefaciens* strain GV3101. Arabidopsis Col-0 was transformed by floral dip as described previously (Clough & Bent, 1998). T1 seeds were germinated in soil and the transformants were BASTA selected. T2 seeds of BASTA-resistant lines were plated on MS media containing 20 μg ml^−1^ phosphinothricin. The lines showing a single insertion based on a 3 : 1 segregation ratio of live : dead seedlings were selected, and homozygous progeny of these plants were used for further quantitative analyses.

### RT-PCR of SAP11 deletion mutants

In order to confirm expression of transgenes in T1 transgenic Arabidopsis, three Arabidopsis leaves were snap frozen and used for RNA extraction with TRI® reagent (Sigma-Aldrich) and purified using Qiagen RNeasy® columns (Qiagen). cDNA was synthesized from 0.5 μg of total RNA using M-MLV reverse transcriptase (Invitrogen). The synthesized cDNA was diluted with distilled water 10-fold and 1 μl was used for RT-PCR using primers attB1 and attB2rev-eGFP for GFP; attB1 and Sap11NLS3′R for SAP11 and SAP11ΔN; Sap11NLS5′F and attB2 for SAP11ΔC and SAP11ΔCΔcc mutants. Go Taq® DNA polymerase was used for these reactions.

### qRT-PCR

The expression levels of SAP11 derivatives in Arabidopsis were quantified by harvesting and snap freezing the aerial part of four 10-d-old T3 homozygous Arabidopsis seedlings grown on Murashige and Skoog (MS) media. cDNA from each sample was prepared as described above and subjected to qRT-PCR using SYBR® Green JumpStart™ Taq ReadyMix™ (Sigma-Aldrich) in a DNA Engine Opticon 2 (BioRad) using the gene-specific primers for SAP11 and Actin 2 (AT3G18780) shown in Table S1. Each reaction was triplicated, and average threshold cycle (*C*_t_) data were used to determine the relative expression levels of SAP11 derivatives compared to Actin 2 gene using the Δ*C*_t_ method.

### Confocal microscopy

SAP11 derivatives cloned in pB7WGF2 (Karimi *et al*., 2002) or eGFP cloned in pB7WG2 (Karimi *et al*., 2002) were transformed into *A. tumefaciens* strain GV3101. The bacteria were cultured overnight in LB medium with 50 μg ml^−1^ of rifampicin and 100 μg ml^−1^ spectinomycin and resuspended in 10 mM MgCl_2_. The culture was diluted to an optical density at 600 nm (OD_600_) of 0.5 and acetosyringone (final concentration of 100 μM) was added. Two leaves of two young *N. benthamiana* plants at the four- to six-leaf stage were pressure infiltrated with the *A. tumefaciens* suspension and left for 3 d.

TCP2 and TCP13 cloned in pB7WGF2 were likewise expressed in *N. benthamiana* plants via agroinfiltration of an *A. tumefaciens* culture diluted to an OD600 of 0.8, as described above. Controls included wild-type SAP11 in pB7WGF2 and eGFP in pB7WG2, infiltrated with an *A. tumefaciens* culture diluted to an OD600 of 0.4.

In order to visualize plant nuclei, 500 ng ml^−1^ to 5.0 μg ml^−1^ of 4′,6-diamidino-2-phenylindole (DAPI) suspended in PBS buffer was pressure infiltrated on the agrobacteria-infiltrated leaves after the 3 d, 2–3 h before visualization using Zeiss 510 Meta laser scanning confocal microscope (Carl Zeiss Ltd, Hertfordshire, UK).

### Yeast two-hybrid analysis

The fragments of SAP11 derivatives and Arabidopsis TCP13 (Sugio *et al*., 2011b) cloned in pDONR207 were cloned into pDEST-GBKT7 and pDEST-GADT7 (Rossignol *et al*., 2007) by LR clonase® II (Invitrogen) and transformed into *Saccharomyces cerevisiae* strain Y187 or AH109 (Matchmaker III; Clontech Laboratories, Mountain View, CA, USA). The interaction studies were carried out by mating compatible strains of yeast, and selection on media depleted of leucine, tryptophan and histidine and on liquid media were performed following Clontech’s instructions and recommendations. To confirm expression of fusion proteins in yeast, 5 ml of overnight culture of yeast cells were incubated in 0.1 M NaOH for 6 min, spun down, resuspended in 50 μl of 4× NuPAGE LDS sample buffer (Invitrogen) and 10 μl of the resuspension was subjected to Western blot analysis. The fusion proteins were detected by monoclonal antibodies against the haemagglutinin antigen HA and c-Myc epitope tags produced in mouse (Sigma-Aldrich).

### Western blotting

Samples were mixed with 4× NuPAGE LDS sample buffer (Invitrogen) and separated on 12.5% (w/v) sodium dodecyl sulfate (SDS) polyacrylamide gels (PAGE) and transferred to 0.45 μm Protran BA85 nitrocellulose membranes (Whatman®, Dassel, Germany) using the Bio-Rad minigel and blotting systems following standard procedures (Sambrook *et al*., 1989). The western blots were incubated with appropriate first antibody and peroxidase-conjugated anti-rabbit or mouse IgG (Sigma-Aldrich) and detection of bound antibodies was conducted with Immobilon Western Chemiluminescent HRP Substrate (Millipore, Watford, UK).

### Coexpression assays

SAP11 derivatives were cloned in pB7WGF2 (Karimi *et al*., 2002), which realizes the expression of N-terminal eGFP fusion protein, and TCP2 and 13 were cloned in pGWB18 (Nakagawa *et al*., 2007), which realizes the expression of N-terminal 4 × c-Myc fusion proteins *in planta*. The constructs were transformed into *Agrobacterium* strain GV3101. The transformants were cultured overnight and resuspended in 10 mM MgCl_2_. The culture was diluted to an optical density at 600 nm (OD_600_) of 0.5. Equal volumes of *A. tumefaciens* suspensions containing appropriate constructs were mixed and acetosyringone (final concentration of 100 μM) was added. The mixtures of *A. tumefaciens* suspensions were infiltrated into *N. benthamiana* plants at the six-leaf stage. Two leaf discs of 1.8 cm (for TCP2 expression shown in Fig.5a) or 1.2 cm (for TCP13 expression shown in Fig.5b) in diameter were ground in 300 μl of extraction buffer (50 mM sodium phosphate, pH 7.0, 10 mM Triton X-100, 10 mM N-lauroylsarcosine, 1 mM 2-mercaptoethanol). Twelve microlitres of the extracts were mixed with 4× NuPAGE LDS sample buffer (Invitrogen) and subjected to western blot. SAP11 derivatives and TCPs were detected by rabbit polyclonal antibodies against full length GFP and c-Myc (A14) epitope (Santa Cruz Biotechnology Inc., Heidelberg, Germany).

For treatment with epoxomicin, *N. benthamiana* leaves were infiltrated with synthetic epoxomicin (Merck Chemicals Ltd, Nottingham, UK) suspended in 100% DMSO (stock concentration of 18 mM) and further diluted in water to a final concentration of 40 μM. To inhibit protease activity, a protease inhibitor cocktail (Sigma Aldrich) was diluted to 1× concentration in water before infiltration. The inhibitors were infiltrated into leaves using a needleless syringe 4 d following agroinfiltration of the same area with *A. tumefaciens* cultures containing either eGFP in pB7WG2 or SAP11 in pB7WGF2 (OD600 of 0.1) and either TCP2 or TCP13 in pGWB18 (OD600 of 0.5). DMSO-containing controls were infiltrated alongside inhibitors and consisted of 100% DMSO suspended in water to an equivalent concentration of inhibitor solutions. Leaf discs were harvested 8 h following treatment with inhibitors or DMSO controls.

### Statistical analyses

All the statistical analysis was completed in Genstat v13 (International Ltd, Hemel Hempstead, UK). Normal distributions of the datasets were examined and Analysis of Variance was conducted. Significance between data was assessed on the basis of a *P* value of < 0.05 by using the Tukey’s multiple comparison test for post-ANOVA analysis.

## Results

### SAP11 N and C-terminal regions are required for the induction of the Arabidopsis leaf crinkling phenotype

**Figure 1:**
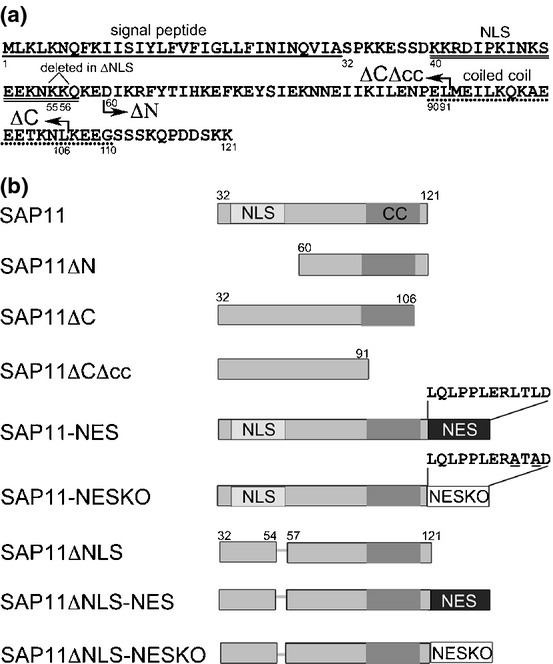
Schematic representation of AY-WB phytoplasma SAP11 virulence effector mutants used in this study. (a) AY-WB phytoplasma SAP11 amino acid sequence. The signal peptide (SP) sequence is underlined with a single line, the bipartite nuclear localization sequence (NLS) with a double line and the predicted coiled coil domain with a dashed line. The numbers below the amino acids refer to those depicted in (b) of this figure and the arrows show the start point of SAP11ΔN and end points of SAP11ΔC and SAP11ΔCΔcc mutants, which did not include the signal peptide sequence. The deletion of two lysines at positions 55 and 56 in the SAP11ΔNLS sequence is shown. (b) Schematic representations of the deletions and addition to SAP11 protein sequence. The light gray squares with ‘NLS’ indicate the location of NLS sequence highlighted in (a) and the dark gray squares with ‘cc’ indicates the coiled coil domain highlighted in (a). The deletion of two lysines at positions 55 and 56 in the SAP11ΔNLS sequence is shown as a line connecting the squares. Some SAP11 constructs have a nuclear export signal (NES) and a defective NES (NESKO), which has two leucines replaced by alanines, fused to the C-termini.

**Figure 2:**
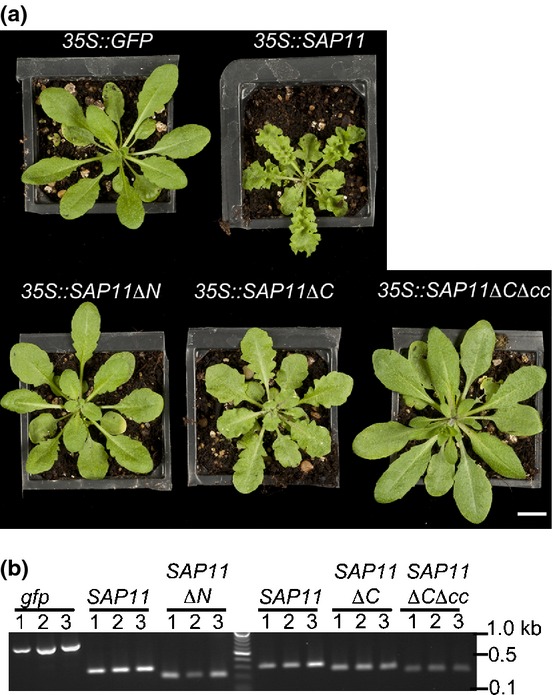
The AY-WB phytoplasma SAP11 N and C terminal regions are required for the induction of leaf crinkling phenotype in Arabidopsis. (a) Transgenic Arabidopsis T1 transgenic plants for GFP, SAP11 and SAP11 mutants expressed under control of the 35S promoter. The transformants were selected by BASTA resistance. Plants shown are 6 wk old. Bar, 1 cm. (b) RT-PCR shows the expression of the transgenes in the T1 transgenic lines shown in (a) of this figure (samples marked as ‘1’ of each line) and two other lines randomly chosen from the T1 transformants (Supporting Information Fig. S2). cDNA was generated from three leaves of the plants shown in the photos. The GFP transgene was amplified with attB1 and GFP-R primers, the SAP11 gene with attB1 and SAP11-3′rev primers (left of marker lane) or SAP11-5′for and attB2 primers (right of marker lane), the *SAP11ΔN* transgene with attB1 and SAP11-3′rev primers, and the *SAP11ΔC* and *SAP11ΔCΔcc* transgenes with SAP11-5′for and attB2 primers.

The SAP11 N-terminal region contains a bipartite nuclear localization signal (NLS) (Figs1, S1) that was previously shown to be required for nuclear targeting of SAP11 (Bai *et al*., 2009). Bioinformatics analysis using the COILS program (Lupas *et al*., 1991) predicted a coiled coil domain in the C-terminal region of SAP11 (Figs1, S1). To dissect the roles of these SAP11 regions, three SAP11 deletion mutants were generated. These are SAP11ΔN lacking the N-terminal portion of SAP11 including the NLS, and SAP11ΔC and SAP11ΔCΔcc lacking the C-terminus up to the coiled coil domain plus four amino acids and the entire coiled coil domain, respectively (Figs1, S1). Stable transgenic Arabidopsis lines that constitutively express the *SAP11ΔN* and *SAP11ΔCΔcc* mutant genes under control of the *Cauliflower mosaic virus* 35S promoter showed wild-type leaf phenotypes similar to those of control *35S::GFP* transgenic lines, whilst leaves of the *35S::SAP11ΔC* plants exhibited clear leaf serration and crinkling similarl to leaves of the *35S::SAP11* plants (Fig.2a, Table1). The expression of the transgenes was confirmed by RT-PCR (Fig.2b). Twelve primary individual transgenic lines for these constructs showed similar leaf phenotypes (Fig. S2). Thus, SAP11 regions between amino acid residues 32–59 and 92–106 (Fig.1) appear to be required for the SAP11-mediated induction of leaf developmental phenotypes.

**Table 1:** Phenotype summary of AY-WB phytoplasma effector SAP11 and derivatives

Mutant name	Plant phenotype (crinkled leaves)	Subcellular localization	TCP binding	TCP destabilization
SAP11	+++	Nucleus	Yes	Yes
SAP11ΔN	+/−	Nucleus and cytoplasm	Yes	No
SAP11ΔC	+	Nucleus	Yes	Yes
SAP11ΔCΔcc	+/−	Nucleus	No	No
SAP11-NES	nd	Nucleus and cytoplasm	nd	nd
SAP11-NESKO	++	Nucleus	Yes	Yes
SAP11ΔNLS	+	Nucleus and cytoplasm	nd	nd
SAP11ΔNLS-NES	+/−	Cytoplasm	Yes	Yes
SAP11ΔNLS-NESKO	nd	Nucleus and cytoplasm	nd	nd

nd, experiment not done.

### The SAP11 N-terminal region is required for nuclear localization

**Figure 3:**
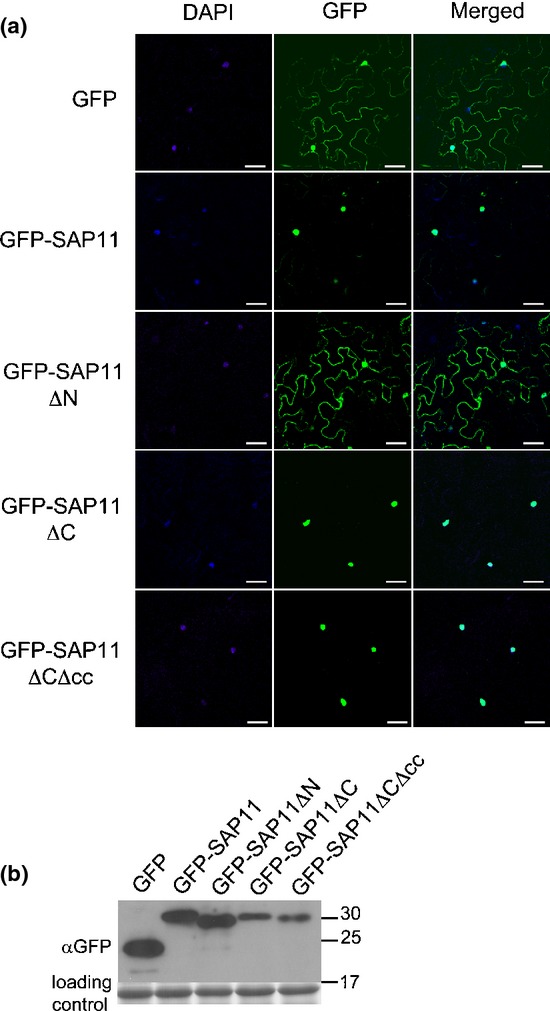
The N-terminal half of the AY-WB phytoplasma mature SAP11 is required for SAP11 localization in plant cell nuclei of *Nicotiana benthamiana* leaves. (a) Confocal microscopy images showing nuclear localization of GFP-SAP11, GFP-SAP11ΔC and GFP-SAP11ΔCΔcc and cytoplasmic presence of GFP-SAP11ΔN and GFP. Leaves of *N. benthamiana* were agroinfiltrated with the various constructs and examined under the confocal microscope after 3 d. Samples were stained with DAPI to indicate positions of cell nuclei. Merged images show overlay of GFP and DAPI fluorescence. Bars, 50 μm. (b) Western blot hybridizations with αGFP IgG showing presence of full-length proteins for all GFP-SAP11 and GFP-SAP11 mutants. Samples for the confocal microscopy and western blots were derived from the same plants and harvested simultaneously. Loading control is Coomassie-stained Ribulose-1,5-bisphosphate carboxylase oxygenase large subunit. Molecular weight markers (kDa) are indicated to the left of the blots.

It is likely that SAP11ΔN does not target plant cell nuclei because this mutant lacks the NLS. To confirm this we transiently produced GFP-fused versions of SAP11ΔN, SAP11ΔC and SAP11ΔCΔcc in *Nicotiana benthamiana* leaves by agroinfiltration and examined the subcellular localization patterns of the GFP fusions. As expected, GFP-SAP11ΔC and GFP-SAP11ΔCΔcc localized primarily to plant cell nuclei similarly to GFP-SAP11, whilst GFP-SAP11ΔN localized in both nuclei and cytoplasm similarly to GFP (Fig.3a, Table1). Full-length fusion proteins were detected in the leaf samples used for microscopy (Fig.3b, Table S2). Thus, the SAP11 N-terminal region is required for subcellular localization in plant cell nuclei and deletions in the C-terminal SAP11 regions do not affect SAP11 targeting to plant cell nuclei.

### The SAP11 C-terminal coiled coil domain binds Arabidopsis TCP13

**Figure 4:**
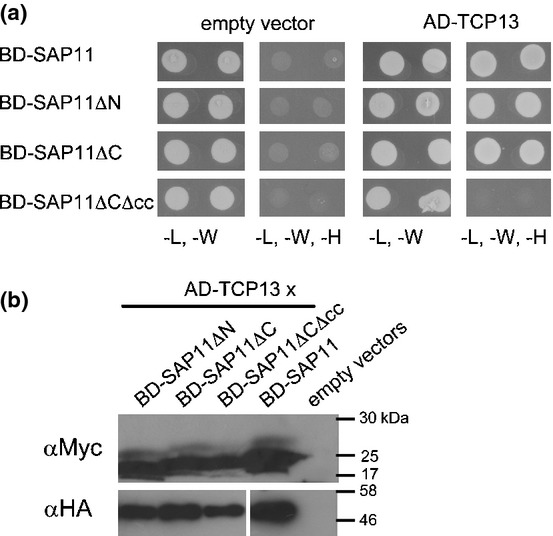
The coiled coil domain of AY-WB phytoplasma SAP11 is required for SAP11 interaction with Arabidopsis TCP13 in yeast. (a) SAP11 and SAP11 mutants were fused at the N-termini to the DNA binding domain (BD) of the GAL4 transcriptional activator (baits) and TCP13 was fused at the N-terminus to the transcription activation domain (AD) of GAL4 (prey). Two independent yeast colonies for each combination of bait or prey or empty plasmid (pDEST-GADT7) were grown in Synthetic dropout (SD) media lacking leucine and tryptophan (-L, -W) or leucine, tryptophan and histidine (-L, -W, -H); the latter indicating interactions of bait and prey. (b) Western blot hybridizations showing the presence of bait and prey in yeast colonies of (a). BD-SAP11 derivatives were detected by αMyc IgG and AD-TCP13 fusion proteins with αHA IgG. Molecular weight markers (kDa) are indicated to the right of the blots.

We conducted yeast two-hybrid analyses to identify the SAP11 regions involved in TCP binding. We used TCP13 (a class II CIN-TCP) as a proxy for SAP11-binding to multiple CIN-TCPs (Sugio *et al*., 2011b) in which SAP11 and derivatives were fused to the DNA binding domain (BD) of the GAL4 transcriptional activator and TCP13 to the transcription activation domain (AD) of this activator. TCP13 shows autoactiviation activity when expressed in the BD construct, a phenomenon commonly observed for transcription factors, and thus we were unable to test the reciprocal arrangement. We observed that BD-SAP11ΔN and BD-SAP11ΔC interacted with AD-TCP13, whilst BD-SAP11ΔCΔcc did not (Fig.4a, Table1). Western blot analysis of yeast extract showed that all the fusion proteins were expressed (Fig.4b). Coiled coil domains are often involved in oligomerization of proteins. To examine if SAP11 forms oligomers, interactions between BD-SAP11 and AD-SAP11 constructs were also examined. BD-SAP11 interacted with AD-TCP13, but not with itself despite abundant presence of proteins in the yeast cells (Fig. S3). Thus, the coiled coil domain is required for SAP11 interaction with TCP13 and is not involved in SAP11 oligomer formation.

### SAP11 N- and C-terminal regions are required for TCP destabilization

**Figure 5:**
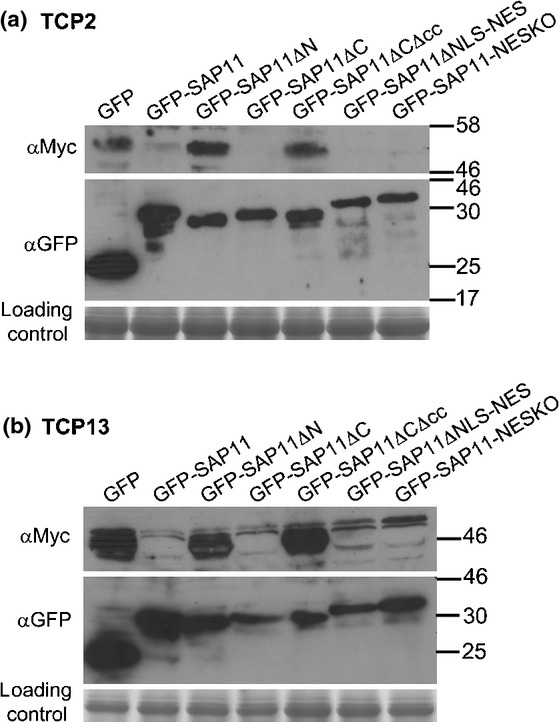
AY-WB phytoplasma SAP11 N and C-terminal regions are required for Arabidopsis TCP2 and TCP13 destabilization in *Nicotiana benthamiana* leaves. Co-agroinfiltration of GFP, GFP-SAP11 and GFP-SAP11 mutants with Myc-tagged TCP2 (a) and Myc-tagged TCP13 (b) in *N. benthamiana* leaves. Full length GFP-SAP11 and GFP-SAP11 mutants were detected with αGFP IgG and Myc-tagged TCP2 or TCP13 were detected with αMyc IgG. The experiments were repeated 3 to 4 times with similar results. The loading control is Coomassie-stained Ribulose-1,5-bisphosphate carboxylase oxygenase large subunit. Molecular weight markers (kDa) are indicated to the right of the blots.

SAP11 interaction with CIN-TCPs results in the destabilization of these transcription factors in *N. benthamiana* leaves using *Agrobacterium tumefaciens* mediated co-expression assays (Sugio *et al*., 2011b). Therefore, these co-expression assays were used to examine TCP destabilization by the SAP11 deletion mutants. SAP11 and derivatives were fused at the N-termini to GFP, and TCP2 and TCP13 at the N-termini to 4 × Myc tags. TCP2 and TCP13 were not destabilized in the presence of GFP alone, whilst fewer or no TCPs were detected in the presence of SAP11 (Fig.5, Tables1, S3, S4) consistent with previous data (Sugio *et al*., 2011b). The TCPs were also destabilized in the presence of GFP-SAP11ΔC, but the transcription factors remained stable in the presence of GFP-SAP11ΔN and GFP-SAP11ΔCΔcc (Fig.5, Tables1, S3, S4). Thus, whilst GFP-SAP11ΔN can bind TCP13 (Fig.4), this SAP11 mutant did not destabilize TCPs. The co-expression experiments were repeated four times with similar results. Together these results indicate that different regions are involved in TCP binding and destabilization and are in agreement with the finding that *35S::SAP11ΔN* and *35S::SAP11ΔCΔcc* transgenic Arabidopsis plants do not show altered leaf development, whilst *35S::SAP11ΔC* transgenic plants do (Fig.2). Treatments with the 26S proteasome inhibitor epoxomicin or protease inhibitor cocktail did not inhibit SAP11-mediated TCP destabilization (Fig. S4) suggesting that TCPs are probably not degraded via the 26S proteasome, SAP11 does not have protease activity and TCPs are degraded by a plant pathway that is insensitive to the protease inhibitor cocktail.

### SAP11 targeting of Arabidopsis nuclei is required for destabilization of CIN-TCPs

**Figure 6:**
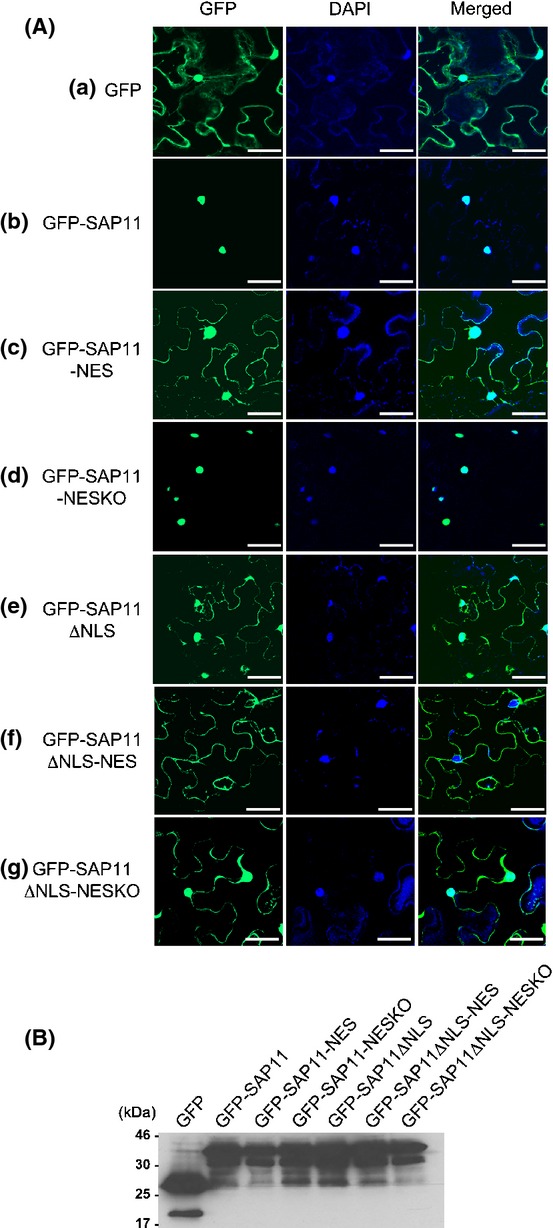
AY-WB phytoplasma SAP11 mutants fused to NES and dysfunctional NLS locate predominantly in plant cell cytoplasm of *Nicotiana benthamiana* leaves. (A) Confocal microscopy images showing subcellular distribution of (a) GFP, (b) GFP-SAP11, (c) GFP-SAP11-NES, (d) GFP-SAP11-NESKO, (e) GFP-SAP11ΔNLS, (f) GFP-SAP11ΔNLS-NES, and (g) GFP-SAP11ΔNLS-NESKO. Leaves of *N. benthamiana* were agroinfiltrated with constructs as shown and examined under the confocal microscope after 3 d. Samples were stained with DAPI to indicate positions of cell nuclei. Merged images show overlay of GFP and DAPI fluorescence. Bars, 50 μm. (B) Western blot hybridizations with αGFP IgG showing the presence of full-length proteins for all GFP-SAP11 and GFP-SAP11 mutants. Samples for the confocal microscopy and western blots were derived from the same plants and harvested simultaneously. Molecular weight markers (kDa) are indicated to the left of the blots.

The finding that SAP11ΔN interacts with TCPs but does not destabilize these transcription factors suggests the possibility that SAP11 nuclear localization is required for destabilization of TCPs, because the NLS is located in the N-terminal region that is deleted from SAP11ΔN. To investigate this possibility, two lysine residues in position 55 and 56 of SAP11 that form a part of the NLS were deleted to create mutant SAP11ΔNLS (Fig.1). GFP-SAP11ΔNLS is localized throughout the cell (Fig.6A(e), Table1) in a similar fashion to GFP (Fig.6A(a)) indicating that the 2 amino-acid deletion in the NLS disrupts SAP11 nuclear targeting. However, that GFP-SAP11ΔNLS is still detected in plant cell nuclei is likely because of passive diffusion from the cytoplasm into nuclei, which is in agreement with the size exclusion limit of the nuclear pore complex being higher than the predicted molecular weight of 38 kDa of GFP-SAP11ΔNLS (27 (Mw of GFP) +11 kDa (Mw of SAP11)) (Gorlich, 1998; Talcott & Moore, 1999). To mostly exclude SAP11 from plant cell nuclei, a nuclear export signal (NES) of HIV–Rev (Fischer *et al*., 1995) was attached to the C-termini of SAP11ΔNLS and SAP11, generating SAP11ΔNLS-NES and SAP11-NES, respectively (Fig.1). As controls, fusions of SAP11ΔNLS and SAP11 to nonfunctional NES (NESKO) generating SAP11ΔNLS-NESKO and SAP11-NESKO, respectively, were analyzed (Fig.1). GFP-SAP11-NES showed increased cytoplasmic distribution compared to GFP-SAP11 (Fig.6A(c), Table1) whilst GFP-SAP11-NESKO was localized mainly to nuclei (Fig.6A(d), Table1) indicating that the NES is functional and the attachment of small peptide (NESKO) does not interfere with subcellular localization of SAP11 mediated by the endogenous NLS. GFP-SAP11ΔNLS-NES was not detected in plant cell nuclei (Fig.6A(f), Table1) whilst GFP-SAP11ΔNLS-NESKO was distributed in both nuclei and cytoplasm (Fig.6Ag, Table1), suggesting that the combined effect of a dysfunctional NLS and a functional NES leads to SAP11 localization predominantly in the plant cell cytoplasm. Full-length versions of GFP-SAP11 fusions and all derivatives were present in infiltrated *N. benthamiana* leaves used for microscopy (Fig.6B).

**Figure 7:**
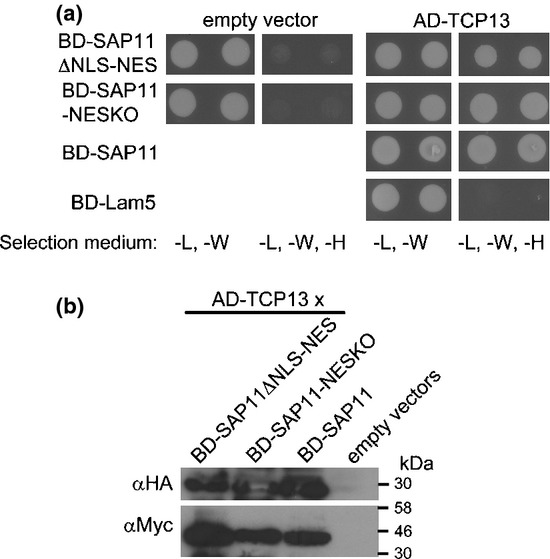
AY-WB phytoplasma SAP11 mutants fused to NES and dysfunctional NLS interact with TCP13 in yeast. (a) SAP11 NLS mutants and NES fusions were fused at the N-termini to the DNA binding domain (BD) of the GAL4 transcriptional activator (bait) and TCP13 was fused at the N-terminus to the transcription activation domain (AD) of GAL4 (prey). BD-SAP11 and BD-Lam5 (human lamin C) served as positive and negative controls, respectively. Two independent yeast colonies for each combination of prey and bait or empty plasmid (pDEST-GADT7) were grown in Synthetic dropout (SD) media lacking leucine and tryptophan (-L, -W) or leucine, tryptophan and histidine (-L, -W, -H); yeast growth on the latter indicated interactions of bait and prey. (b) Western blot hybridizations showing the presence of bait and prey in yeast colonies of A. BD-SAP11 derivatives were detected by αMyc IgG and AD-TCP13 fusion proteins with αHA IgG. Molecular weight markers (kDa) are indicated to the right of the blots.

The SAP11 NLS and NES mutants were analysed for their ability to interact with and destabilize TCPs. BD-SAP11ΔNLS-NES and BD-SAP11-NESKO interacted with AD-TCP13 in the yeast two-hybrid assay (Fig.7, Tables1, S5) although cells with BD-SAP11ΔNLS-NES and AD-TCP13 constructs were growing slower than those with the BD-SAP11-NESKO and AD-TCP13 constructs and BD-SAP11 and AD-TCP13 constructs. It is possible that BD-SAP11ΔNLS-NES is exported out of the nucleus and requires presence in yeast cell nuclei to interact with AD-TCP13 and induce the His3 reporter gene. Co-expression analyses in *N. benthamiana* leaves revealed that GFP-SAP11ΔNLS-NES and GFP-SAP11-NESKO destabilized TCP2 and TCP13 (Fig.5, Tables1, S3, S4). Thus, the ΔNLS mutation and the NES fusion do not affect the ability of SAP11 to interact and destabilize TCPs.

**Figure 8:**
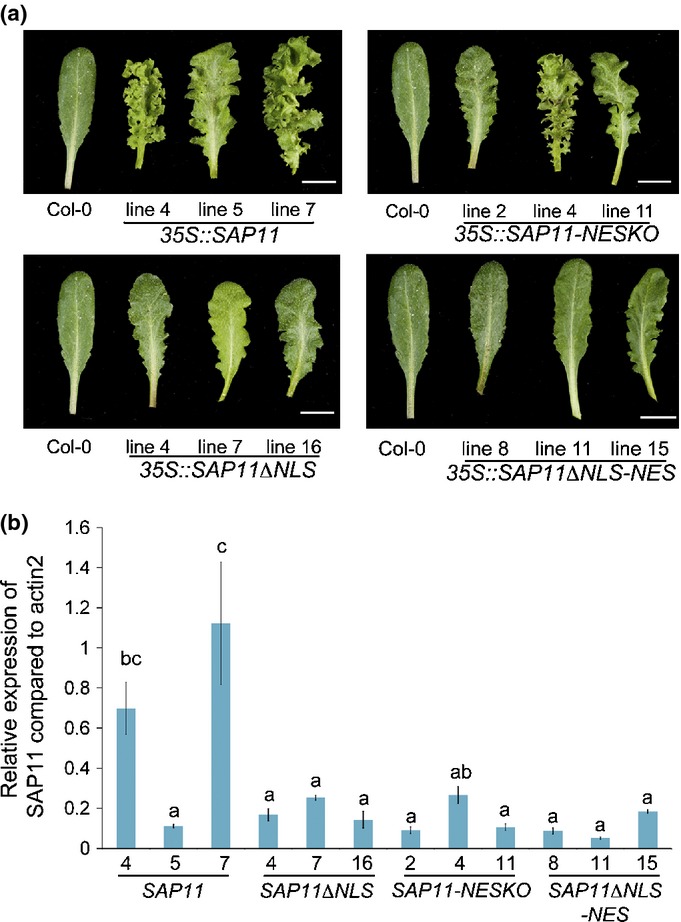
AY-WB phytoplasma SAP11 fused to NES and dysfunctional NLS have reduced ability to induce leaf crinkling in Arabidopsis. (a) Photographs of representative leaves were taken from 10-wk-old T3 Arabidopsis homozygous lines expressing the transgenes under control of the 35S promoter. The plants were grown in a short day conditions (8 h : 16 h, light : dark). Bars, 1 cm. (b) Relative expression levels compared to actin2 gene of transgenes of transgenic lines shown in (a) Columns present transgene expression levels and standard errors (SEs) (bars) of qRT-PCRs from three biological replicates. Different letters (a–c) above the columns indicate statistical differences (*P* < 0.05) calculated by ANOVA and Tukey’s multiple comparison test.

The NLS mutants and NES fusions of SAP11 were expressed under control of the 35S promoter in Arabidopsis. Three independent T3 homozygous lines were selected and analysed for transgene expression levels. The expression levels of SAP11ΔNLS and NES mutant transgenes were equal or higher than that of the SAP11 expression level of *35S::SAP11* line 5 (Figs8, S5), which has an obvious leaf serration and crinkling phenotype (Figs8, S5, Table1) (Sugio *et al*., 2011b). The *35S::SAP11-NESKO* lines exhibited similar leaf crinkling phenotypes as the 35S::SAP11 lines (Figs8, S5 and Table1) indicating that that NES fusion does not interfere with SAP11 activity in the transgenic plants. The *35S::SAP11ΔNLS* and *35S::SAP11ΔNLS-NES* lines both had reduced leaf serration and crinkling phenotypes that were almost comparable to wild-type Col-0 leaves. Taken together, these results suggest that the NLS is not involved in the direct interaction of SAP11 with TCPs and TCP destabilization, but that SAP11 localization to nuclei is required for efficient TCP destabilization in Arabidopsis. This is in agreement with the nuclear localization of TCP2 and TCP13 (Fig. S6) (Baba *et al*., 2001; Suzuki *et al*., 2001; Koroleva *et al*., 2005; Martin-Trillo & Cubas, 2010), indicating that SAP11 likely interacts with and possibly destabilizes its targets in the nucleus.

## Discussion

We found that SAP11 mutants lacking the entire N-terminal domain, including the NLS, interacted with TCPs but were impaired in the destabilization of these transcription factors. In addition, SAP11 mutants that lacked the C-terminal domain, including a predicted coiled coil structure, were impaired in both binding and destabilization of TCPs. Unlike wild-type SAP11, these SAP11 mutants did not alter leaf morphogenesis. SAP11 mutants with mutations in the NLS and with a nuclear export signal (NES) had cytoplasmic distributions and both bound and destabilized TCP transcription factors, but instigated weaker changes in Arabidopsis leaf morphogenesis than wild-type SAP11. Whilst the various deletions may affect the three-dimensional structure of SAP11, our data suggest that this structure may not be essential for the three SAP11 activities. First, deletion of the N-terminal 28 amino acid of the mature (without signal peptide) 90-amino acid SAP11 protein did not affect TCP binding of SAP11 and deletion of C-terminal 30 amino acid did not affect SAP11 nuclear localization. Secondly, SAP11 mutants without the C-terminal 15 amino acids bind and destabilize TCPs. Finally, mutations in the NLS or addition of a NES did not affect SAP11 TCP binding and destabilization. Thus, SAP11 appears to have a modular organization in which specific amino acids/domains in different parts of the SAP11 protein are required for nuclear localization, TCP binding or destabilization, and these domains appear to not affect each other’s activities. A linear modular structure of SAP11 is in agreement with the hypothesis that additions of GFP, NES and other tags to the SAP11 N- and C-termini do not compromise the SAP11 ability of nuclear localization and TCP binding and destabilization.

We demonstrated that SAP11 interaction with TCPs is not sufficient for TCP destabilization and induction of leaf crinkling, because SAP11ΔN – which binds but not destabilizes CIN-TCPs – does not induce leaf crinkling in Arabidopsis. Therefore, it is unlikely that SAP11 blocks TCP action through steric hindrance, but rather mediates active degradation of CIN-TCPs, possibly by interacting with a plant-specific helper component that has a role in the plant protein degradation pathway. The involvement of a plant helper component is also suggested by the yeast two-hybrid experiments in which the TCPs were not degraded in the presence of wild-type SAP11 and SAP11ΔN. Given that we did not observe a reduction of destabilization in the presence of proteasome inhibitor and protease inhibitor cocktail, it remains unclear how SAP11 mediates destabilization of TCPs complicating studies of where and how SAP11-mediated TCP degradation occurs in the cell. The mechanism may be revealed as soon as a plant helper component is identified. We predict that this helper protein binds to the SAP11 N-terminal 28 amino acids in the mature protein as this domain is required for TCP destabilization but not TCP binding.

Given the important function of CIN-TCPs in leaf morphogenesis, we used the amount of leaf crinkling as a proxy for CIN-TCP presence and function. SAP11ΔNLS-NES transgenic Arabidopsis plants show no or reduced leaf crinkling phenotypes compared to SAP11 transgenic plants, indicating that nuclear localization of SAP11 contributes to TCP destabilization. Indeed, many TCPs have mono- or bipartite NLSs and nuclear localization has been demonstrated experimentally (Baba *et al*., 2001; Suzuki *et al*., 2001; Koroleva *et al*., 2005; Martin-Trillo & Cubas, 2010), although some TCPs target chloroplasts (Baba *et al*., 2001). Nonetheless, SAP11ΔNLS-NES is still able to destabilize TCPs in co-expression analyses in *N. benthamiana* leaves. This apparent discrepancy may be due to incomplete exclusion of SAP11ΔNLS-NES from the nucleus. SAP11ΔNLS-NES is a small protein that can passively migrate into the nucleus, but once in the nucleus the NES transports this protein out of the nucleus. In *N. benthamiana* transient assays, where transgenes are expressed at high levels, sufficient amounts of SAP11ΔNLS-NES may migrate into the nucleus to interact with the nuclear-localized TCPs. It is not yet known if degradation of TCPs occurs inside the nucleus or cytoplasm or in both cell compartments. Therefore, nuclear targeting of SAP11 likely increases the opportunity of SAP11 to bind and destabilize CIN-TCPs.

**Figure 9:**
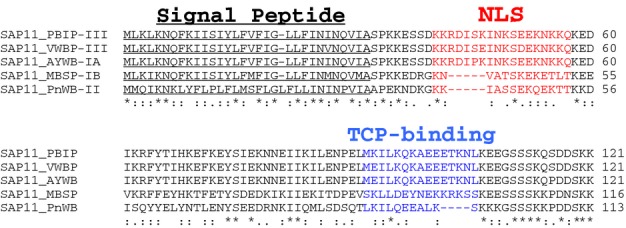
Multiple sequence alignment of AY-WB phytoplasma SAP11 with SAP11 homologs of other phytoplasmas. SAP11 protein sequences obtained from Poinsettia Branch-Inducing Phytoplasma (PBIP), Vaccinium Witches’-Broom Phytoplasma (VWBP), Aster Yellows phytoplasma strain Witches’ Broom (AYWB), Maize bushy stunt phytoplasma (MBSP) and Peanut Witches’ Broom (PnWB) were aligned using the CLUSTAL 2.1 program. PBIP and VWBP belong to 16SrDNA group III, AYWB and MBSP to groups 1A and 1B, respectively, and PnWB to group II as indicated. Genbank accession numbers: GI:515759334 (PBIP); GI:515761117 (VWBP); GI:85057650 (AYWB); GI:471234556 (PnWB). Signal peptide sequence is underlined, nuclear localization signal (NLS) is indicated in red font, and part of coiled coil structure required for binding TCP in blue font. *, Fully conserved residues, :, conservation of residues with strongly similar properties and., conservation of residues with weakly similar properties.

The coiled coil domain located between residues 91 and 106 of SAP11 protein (Fig.1a) is required for SAP11 interactions with the CIN-TCPs. We previously provided evidence that SAP11 interacts with the 59-amino acid basic helix-loop-helix motif TCP domain that is involved in the DNA binding and protein–protein interactions of TCP factors (Cubas *et al*., 1999; Kosugi & Ohashi, 2002; Sugio *et al*., 2011b). Because coiled coil is a structural motif in proteins that allows 2–7 α-helices to coil together (Liu *et al*., 2006), it is likely that SAP11 interacts with one or both of the helices in the TCP domain. Helix 1 is less conserved than helix 2 amongst the class I and class II CIN and CYC/TB1 TCPs (Martin-Trillo & Cubas, 2010), allowing for SAP11-binding specificity amongst the different TCPs. SAP11 virulence effectors with highly similar sequences have been identified in diverse phytoplasmas that belong to evolutionary distinct clusters in the phytoplasma phylogenetic tree (Fig.9) (Hogenhout *et al*., 2008; Chung *et al*., 2013). Intriguingly, alignment of the SAP11 protein sequences reveals that the NLS and TCP-binding coiled coil domain are least conserved (Fig.9) indicating that these regions may be under selection allowing diversity of SAP11 homologs in nuclear vs cytoplasmic targeting and interaction with a different sets of TCPs or other (transcription factor) targets in the plant.

It was noticed that 35S:SAP11 transgenic lines have higher levels of variations in transgene expression levels than the transgenic lines expressing mutants of SAP11 (Fig.8b). A possible explanation is that leaves of SAP11 transgenic lines produce more cells (as evidenced by the curly leaves) and because CIN-TCPs, which are degraded by SAP11, promote cell maturation the cells may also be metabolically more active in the SAP11 transgenic lines than in SAP11 mutant lines. Thus, small changes in initial wild-type SAP11 expression levels may be amplified into larger expression differences during plant growth.

SAP11 is the only virulence effector of phytoplasmas for which plant targets has been identified so far. However, phytoplasmas have multiple virulence effectors; more than 50 candidate virulence effectors has been identified in AY-WB (Bai *et al*., 2009), many of which are represented in different phytoplasmas (Chen *et al*., 2012; Saccardo *et al*., 2012; Chung *et al*., 2013). The majority of the phytoplasma virulence effector genes lie on genetic islands resembling mobile transposons that may have been derived from ancient prophage attacks and that are likely exchanged between phytoplasmas (Bai *et al*., 2006; Jomantiene & Davis, 2006; Wei *et al*., 2008; Chung *et al*., 2013) providing a possible explanation of why SAP11 homologs of distantly related phytoplasmas are more similar in sequence than homologs of more closely related phytoplasmas (Fig.9). Other phytoplasma virulence effectors, such as TENGU and SAP54, also have a region with a predicted coiled coil structure. Whilst the targets of these effectors have not yet been described, they both induce dramatic changes in plant development; TENGU induces witches’ brooms and dwarfism (Hoshi *et al*., 2009; Sugawara *et al*., 2013) and SAP54 the formation of leafy indeterminate flower development (MacLean *et al*., 2011), indicating that these effectors probably target plant (transcription) factors as well. Thus, phytoplasma virulence effectors may have evolved as versatile linear modular proteins that target a variety of plant (transcription) factors to evoke architectural changes in plant hosts. This effector versatility may be particularly important for phytoplasma success, because these bacteria are dependent on insect vectors for dispersal and hence do not choose their plant hosts. If the changes are beneficial to phytoplasma fitness by, for example, generating more plant tissue for colonization by phytoplasmas and the insect vectors that disperse the phytoplasmas (MacLean *et al*., 2011; Sugio *et al*., 2011a,b; Sugio & Hogenhout, 2012), the effector genes are more likely to prevail in phytoplasma populations.

## Supporting Information

**Fig. S1** Prediction of the coiled coil domain in the C-terminal region of the AY-WB phytoplasma virulence effector SAP11. **Fig. S2** Arabidopsis T1 transgenic lines expressing AY-WB phytoplasma SAP11 and SAP11 mutants. **Fig. S3** AY-WB phytoplasma SAP11 does not interact with itself. **Fig. S4** AY-WB phytoplasma SAP11-mediated destabilization of Arabidopsis TCP2 and TCP13 is not inhibited by protease or proteasomal inhibitors in *N. benthamiana* leaves. **Fig. S5** Arabidopsis transgenic lines expressing AY-WB phytoplasma SAP11 and SAP11 mutants. **Fig. S6** Arabidopsis TCP2 and TCP13 and AY-WB phytoplasma SAP11 are localized to the plant cell nuclei in *N. benthamiana* leaves. **Table S1** Primers used in this study **Table S2** Signal intensity levels (ImageJ) of bands in Fig. (b) **Table S3** Signal intensity levels (ImageJ) of bands in Fig. (a) (TCP2) **Table S4** Signal intensity levels (ImageJ) of bands in Fig. (b) (TCP13) **Table S5** Signal intensity levels (ImageJ) of bands in Fig.
