# Supplementary material for: The small phytoplasma virulence effector SAP11 contains distinct domains required for nuclear targeting and CIN-TCP binding and destabilization
Source: New Phytol. 2014 Feb 19;202(3):838–48. doi: 10.1111/nph.12721 (PMC4235307; doi:10.1111/nph.12721)
Supplement: Supplementary file 1 — Fig. S1 Prediction of the coiled coil domain in the C-terminal region of the AY-WB phytoplasma virulence effector SAP11. Fig. S2 Arabidopsis T1 transgenic lines expressing AY-WB phytoplasma SAP11 and SAP11 mutants. Fig. S3 AY-WB phytoplasma SAP11 does not interact with itself. Fig. S4 AY-WB phytoplasma SAP11-mediated destabilization of Arabidopsis TCP2 and TCP13 is not inhibited by protease or proteasomal inhibitors in N. benthamiana leaves. Fig. S5 Arabidopsis transgenic lines expressing AY-WB phytoplasma SAP11 and SAP11 mutants. Fig. S6 Arabidopsis TCP2 and TCP13 and AY-WB phytoplasma SAP11 are localized to the plant cell nuclei in N. benthamiana leaves. Table S1 Primers used in this study Table S2 Signal intensity levels (ImageJ) of bands in Fig. (b) Table S3 Signal intensity levels (ImageJ) of bands in Fig. (a) (TCP2) Table S4 Signal intensity levels (ImageJ) of bands in Fig. (b) (TCP13) Table S5 Signal intensity levels (ImageJ) of bands in Fig. [file nph0202-0838-SD1.pdf]

Supporting information

The small phytoplasma virulence effector SAP11 contains distinct domains required for nuclear targeting and CIN-TCP binding and destabilization

Akiko Sugio, Allyson M. MacLean and Saskia A. Hogenhout

Cell and Developmental Biology, The John Innes Centre, Norwich Research Park, Norwich NR4 7UH, United Kingdom

Contents

Table S1. Primers used in this study.

Table S2. Signal intensity levels (ImageJ) of bands in Fig. 3B.

Table S3. Signal intensity levels (ImageJ) of bands in Fig. 5A (TCP2).

Table S4. Signal intensity levels (ImageJ) of bands in Fig. 5B (TCP13).

Table S5. Signal intensity levels (ImageJ) of bands in Fig. 7.

Fig. S1. Prediction of the coiled coil domain in the C-terminal region of the AY-WB phytoplasma virulence effector SAP11.

Fig. S2. Arabidopsis T1 transgenic lines expressing AY-WB phytoplasma SAP11 and SAP11 mutants.

Fig. S3. AY-WB phytoplasma SAP11 does not interact with itself.

Fig. S4. AY-WB phytoplasma SAP11-mediated destabilization of Arabidopsis TCP2 and TCP13 is not inhibited by protease or proteasomal inhibitors in *N. benthamiana* leaves.

Fig. S5. Arabidopsis transgenic lines expressing AY-WB phytoplasma SAP11 and SAP11 mutants.

Fig. S6. Arabidopsis TCP2 and TCP13 and AY-WB phytoplasma SAP11 are localized to the plant cell nuclei in *N. benthamiana* leaves.

Table S1. Primers used in this study.

primer name	sequence 5'-> 3'
For cloning and RTPCR	
attB1-adapter	GGGGACAAGTTTGTACAAAAAAGCAGGCT
attB2-adapter	GGGGACCACTTTGTACAAGAAAGCTGGGT
attB1sap11dF2	AAAAAGCAGGCTCCACCATGGATATTAAGAGATTTTATACTATTC
FullattBadaptSAP11F	GGGGACAAGTTTGTACAAAAAAGCAGGCTCCACCATGTCTCCAAAGAAGGAATCTTC
FullattB2sap11dR1	GGGGACCACTTTGTACAAGAAAGCTGGGTGTTAAAGATTCTTTGTTTCCTCTTCAGC
FullattB2sap11dR2	GGGGACCACTTTGTACAAGAAAGCTGGGTGTTAAAGCTCTGGATTTTCAAGAATCT
SAP11optNLS3QCF	CAAATCTGAAGAGAAGAACCAAAAAGAGGATATTAAGAG
SAP11optNSL3QCR	CTCTTAATATCCTCTTTTTGGTTCTTCTCTTCAGATTTG
SAP11NESattB2	AGAAAGCTGGGTGTCATTAATCGAGAGTAAGTCTTTCAAGTGGAGGGAGTTGAAGCTTCTTAGAATCATCAGG
SAP11NESKOattB2	AGAAAGCTGGGTGTCATTAATCAGCAGTAGCTCTTTCAAGTGGAGGGAGTTGAAGCTTCTTAGAATCATCAGG
attB2rev-eGFP	AGAAAGCTGGGTGTTACTTGTACAGCTCGTCCATGCCGAG
Sap11NLS5'F	AAAAAGCAGGCTCCACCATGTCTCC
Sap11NLS3'R	AGAAAGCTGGGTCTTACTTCTTAGAATC
attB1	ACAAGTTTGTACAAAAAAGCAGGC
attB2	ACCACTTTGTACAAGAAAGCTGGG
For qRTPCR	
RTSAP11optF2	TTCTTGAAAATCCAGAGCTTATGGA
RTSAP11optR2	CTTCTTAGAATCATCAGGTTGCTTTG
Actin2-newF	GATGAGGCAGGTCCAGGAATC
Actin2-newR	GTTTGTCACACACAAGTGCATC

Table S2. Signal intensity levels (ImageJ) of bands in Fig. 3B

Lane	αGFP	Loading control	Relative intensity
GFP	50103.36	18136.34	2.76
GFP-SAP11	37550.44	19071.80	1.97
GFP-SAP11DN	45053.80	18927.51	2.38
GFP-SAP11DC	18892.89	18879.92	1.00
GFP-SAP11DCDcc	17421.79	18371.09	0.95

Table S3. Signal intensity levels (ImageJ) of bands in Fig. 5A (TCP2)

Lane	αMyc	αGFP	Loading control
GFP	18366.79	26738.74	11568.38
GFP-SAP11	3138.79	18728.06	11169.33
GFP-SAP11DN	29777.32	11602.27	11962.40
GFP-SAP11DC	387.36	9726.44	11310.77
GFP-SAP11DCDcc	21049.48	12572.04	11828.23
GFP-SAP11DNLS-NES	325.78	7581.59	11049.96
GFP-SAP11-NESKO	602.85	7344.57	13647.30

TCP relative intensity (αMyc/loading control)	
GFP	1.59
GFP-SAP11	0.28
GFP-SAP11DN	2.49
GFP-SAP11DC	0.03
GFP-SAP11DCDcc	1.78
GFP-SAP11DNLS-NES	0.03
GFP-SAP11-NESKO	0.04

GFP relative intensity (αGFP/loading control)	
GFP	2.31
GFP-SAP11	1.68
GFP-SAP11DN	0.97
GFP-SAP11DC	0.86
GFP-SAP11DCDcc	1.06
GFP-SAP11DNLS-NES	0.69
GFP-SAP11-NESKO	0.54

Table S4. Signal intensity levels (ImageJ) of bands in Fig. 5B (TCP13)

Lane	αMyc	αGFP	Loading control
GFP	76831.40	49204.30	22933.25
GFP-SAP11	9008.69	45448.88	23273.84
GFP-SAP11DN	57576.67	44676.03	21707.76
GFP-SAP11DC	13204.11	19333.21	20670.06
GFP-SAP11DCDcc	66581.27	23456.98	23906.64
GFP-SAP11DNLS-NES	21894.00	18335.2	24896.52
GFP-SAP11-NESKO	25552.99	31117.78	25128.89

TCP relative intensity (αMyc/loading control)	
GFP	3.35
GFP-SAP11	0.39
GFP-SAP11DN	2.65
GFP-SAP11DC	0.64
GFP-SAP11DCDcc	2.79
GFP-SAP11DNLS-NES	0.88
GFP-SAP11-NESKO	1.02

GFP relative intensity (αGFP/loading control)	
GFP	2.15
GFP-SAP11	1.95
GFP-SAP11DN	2.06
GFP-SAP11DC	0.94
GFP-SAP11DCDcc	0.98
GFP-SAP11DNLS-NES	0.74
GFP-SAP11-NESKO	1.24

Table S5. Signal intensity levels (ImageJ) of bands in Fig. 7

Lane	αHA	αMyc
BD-SAP11DNLS-NES	25616.15	25256.01
BD-SAP11-NESKO	8099.77	13743.99
BD-SAP11	18826.64	14775.82
Empty vectors	2465.40	0

Figure S1. Prediction of the coiled coil domain in the C-terminal region of AY-WB phytoplasma virulence effector SAP11. The prediction of coiled coil structure (highlighted in blue font) was generated in COILS version 1.0. using MTIDK matrix and **weights**: a,d=2.5 and b,c,e,f,g=1.0. Residue number, residue type and the frame and coiled-coil-forming probability obtained in scanning windows of 14, 21 and 28 residues are shown. Starts and ends of SAP11ΔN, SAP11ΔC and SAP11ΔCΔcc (Figure 1) are indicated. The residues that show coiled-coil-forming probability over 0.5 in any one of the frames are shown in blue.

		14		21		28	
1	M	e	0.002	e	0.000	e	0.000
2	L	f	0.002	f	0.000	f	0.000
3	K	g	0.002	g	0.000	g	0.000
4	L	a	0.002	a	0.000	a	0.000
5	K	b	0.002	b	0.000	b	0.000
6	N	c	0.002	c	0.000	c	0.000
7	Q	d	0.002	d	0.000	d	0.000
8	F	e	0.002	e	0.000	e	0.000
9	K	f	0.002	f	0.000	f	0.000
10	I	g	0.002	g	0.000	g	0.000
11	I	a	0.002	a	0.000	a	0.000
12	S	b	0.002	b	0.000	b	0.000
13	I	c	0.002	c	0.000	c	0.000
14	Y	d	0.002	d	0.000	d	0.000
15	L	a	0.002	a	0.000	a	0.000
16	F	e	0.001	e	0.000	e	0.000
17	V	f	0.001	f	0.000	f	0.000
18	F	g	0.001	g	0.000	g	0.000
19	I	a	0.002	a	0.000	a	0.000
20	G	b	0.002	b	0.000	b	0.000
21	L	c	0.002	c	0.000	c	0.000
22	L	d	0.002	d	0.000	d	0.000
23	F	e	0.002	e	0.000	e	0.000
24	I	f	0.002	f	0.000	f	0.000
25	N	g	0.002	g	0.000	g	0.000
26	I	a	0.002	a	0.000	a	0.000
27	N	b	0.002	b	0.000	b	0.000
28	Q	c	0.002	c	0.000	c	0.000
29	V	d	0.002	d	0.000	d	0.000
30	I	e	0.002	e	0.000	e	0.000
31	A	f	0.002	f	0.000	f	0.000
32	S	g	0.002	g	0.000	g	0.000
33	P	f	0.000	f	0.000	f	0.000
34	K	e	0.001	e	0.000	e	0.000
35	K	f	0.001	f	0.000	f	0.000
36	E	g	0.001	g	0.000	g	0.000
37	S	a	0.001	a	0.000	a	0.000
38	S	b	0.001	b	0.000	b	0.000
39	D	c	0.001	c	0.001	c	0.000
40	K	d	0.001	d	0.001	d	0.000
41	K	e	0.001	e	0.001	e	0.000
42	R	f	0.001	f	0.001	f	0.000
43	D	g	0.001	g	0.001	g	0.000
44	I	a	0.001	a	0.002	a	0.000
45	P	f	0.001	f	0.002	f	0.000
46	K	g	0.087	g	0.045	g	0.016
47	I	a	0.087	a	0.045	a	0.016

48	N	b	0.087	b	0.045	b	0.016
49	K	c	0.087	c	0.045	c	0.016
50	S	d	0.087	d	0.045	d	0.016
51	E	e	0.168	e	0.045	e	0.016
52	E	f	0.168	f	0.045	f	0.016
53	K	g	0.168	g	0.045	g	0.016
54	N	a	0.168	a	0.045	a	0.016
55	K	b	0.168	b	0.045	b	0.016
56	K	c	0.168	c	0.045	c	0.016
57	Q	d	0.168	d	0.045	d	0.016
58	K	e	0.168	e	0.045	e	0.016
59	E	f	0.168	f	0.045	f	0.016
ΔN>							
60	D	g	0.168	g	0.045	g	0.016
61	I	a	0.168	a	0.045	a	0.016
62	K	b	0.168	b	0.045	b	0.016
63	R	c	0.168	c	0.045	c	0.016
64	F	d	0.168	d	0.045	d	0.016
65	Y	e	0.017	e	0.045	e	0.016
66	T	f	0.007	f	0.045	f	0.016
67	I	d	0.004	d	0.012	d	0.016
68	H	e	0.004	e	0.004	e	0.016
69	K	f	0.004	f	0.004	f	0.016
70	E	g	0.004	g	0.004	g	0.016
71	F	a	0.004	a	0.004	a	0.016
72	K	b	0.012	b	0.004	b	0.016
73	E	c	0.012	c	0.004	c	0.016
74	Y	d	0.012	d	0.004	d	0.002
75	S	g	0.043	g	0.004	g	0.002
76	I	a	0.043	a	0.004	a	0.002
77	E	b	0.043	b	0.004	b	0.002
78	K	c	0.043	c	0.004	c	0.002
79	N	d	0.043	d	0.004	d	0.005
80	N	e	0.043	e	0.004	e	0.006
81	E	f	0.043	f	0.004	f	0.007
82	I	g	0.043	g	0.004	g	0.007
83	I	a	0.043	a	0.004	a	0.007
84	K	b	0.043	b	0.004	b	0.007
85	I	c	0.043	c	0.004	c	0.007
86	L	d	0.043	d	0.004	d	0.007
87	E	e	0.043	e	0.016	e	0.007
88	N	f	0.043	f	0.016	f	0.007
89	P	b	0.030	b	0.179	b	0.007
90	E	f	0.358	f	0.694	f	0.007
91	L	g	0.358	g	0.694	g	0.007
ΔCΔcc>							
92	M	a	0.549	a	0.694	a	0.007
93	E	b	0.736	b	0.694	b	0.007
94	I	c	0.736	c	0.694	c	0.007
95	L	d	0.951	d	0.694	d	0.007
96	K	e	0.951	e	0.694	e	0.007
97	Q	f	0.951	f	0.694	f	0.007
98	K	g	0.951	g	0.694	g	0.007
99	A	a	0.951	a	0.694	a	0.007
100	E	b	0.951	b	0.694	b	0.007
101	E	c	0.951	c	0.694	c	0.007
102	E	d	0.951	d	0.694	d	0.007
103	T	e	0.951	e	0.694	e	0.007
104	K	f	0.951	f	0.694	f	0.007
105	N	g	0.951	g	0.694	g	0.007
106	L	a	0.951	a	0.694	a	0.007
ΔC>							
107	K	b	0.951	b	0.694	b	0.007
108	E	c	0.951	c	0.694	c	0.007
109	E	g	0.707	g	0.694	g	0.007
110	G	e	0.037	e	0.694	e	0.006
111	S	f	0.018	f	0.433	f	0.006

112	S	g	0.008	g	0.416	g	0.002
113	S	a	0.001	a	0.052	a	0.000
114	K	b	0.001	b	0.052	b	0.000
115	Q	c	0.001	c	0.052	c	0.000
116	P	c	0.000	c	0.000	c	0.000
117	D	f	0.000	f	0.000	f	0.000
118	D	g	0.000	g	0.000	g	0.000
119	S	a	0.000	a	0.000	a	0.000
120	K	b	0.000	b	0.000	b	0.000
121	K	c	0.000	c	0.000	c	0.000

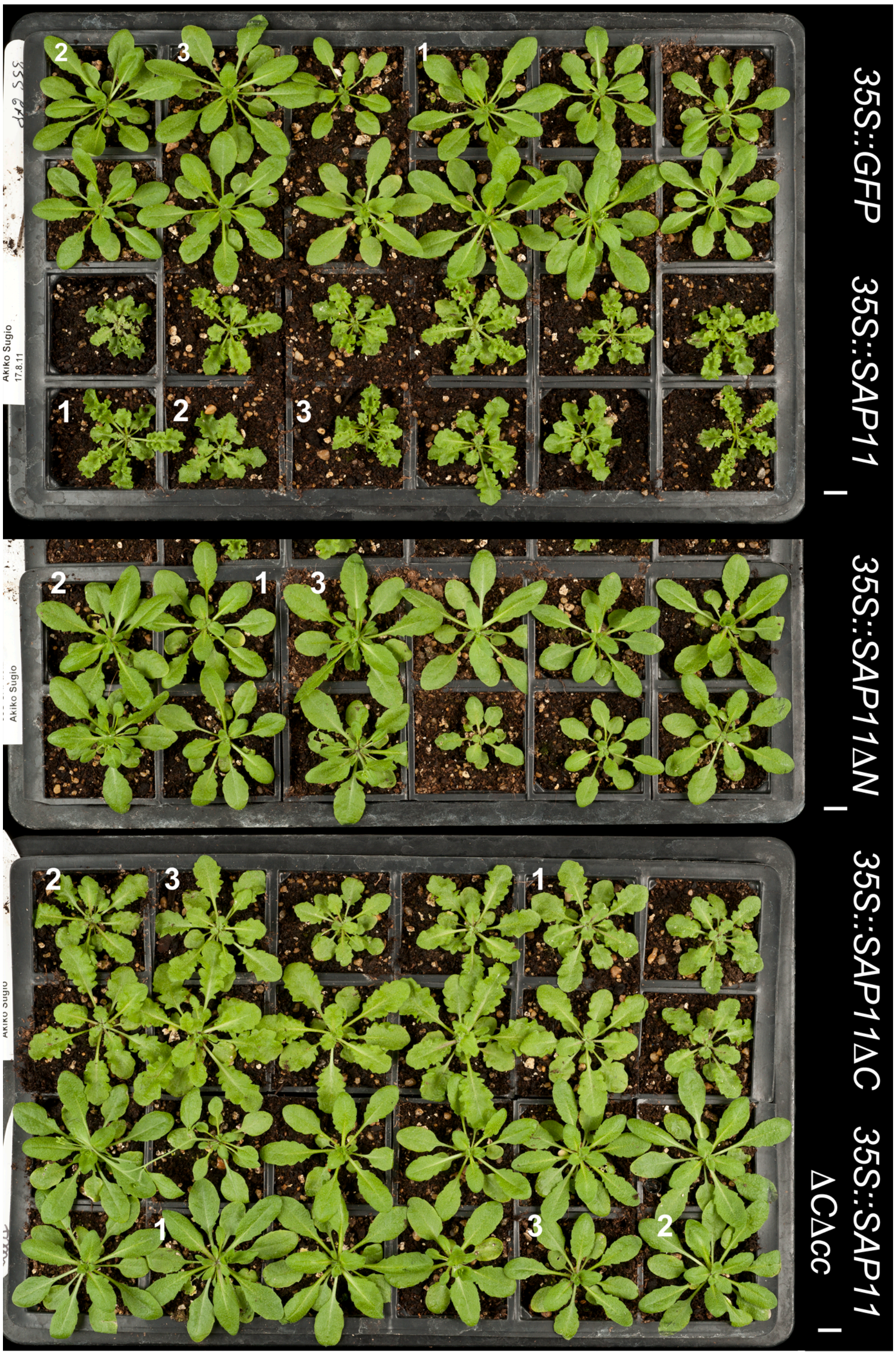

Figure S2. Arabidopsis T1 transgenic lines expressing AY-WB phytoplasma SAP11 and SAP11 mutants. Photos of 12 independent transgenic Arabidopsis T1 transgenic plants for GFP, SAP11 and SAP11 mutants expressed under control of the 35S promoter. Transformants were selected by BASTA resistance. Plants shown are 6 weeks old. Bars=1cm. The plants labelled as "1" were used for the photograph presented in **Figure 2**. Line numbers correspond to the sample numbers in **Figure 2B**.

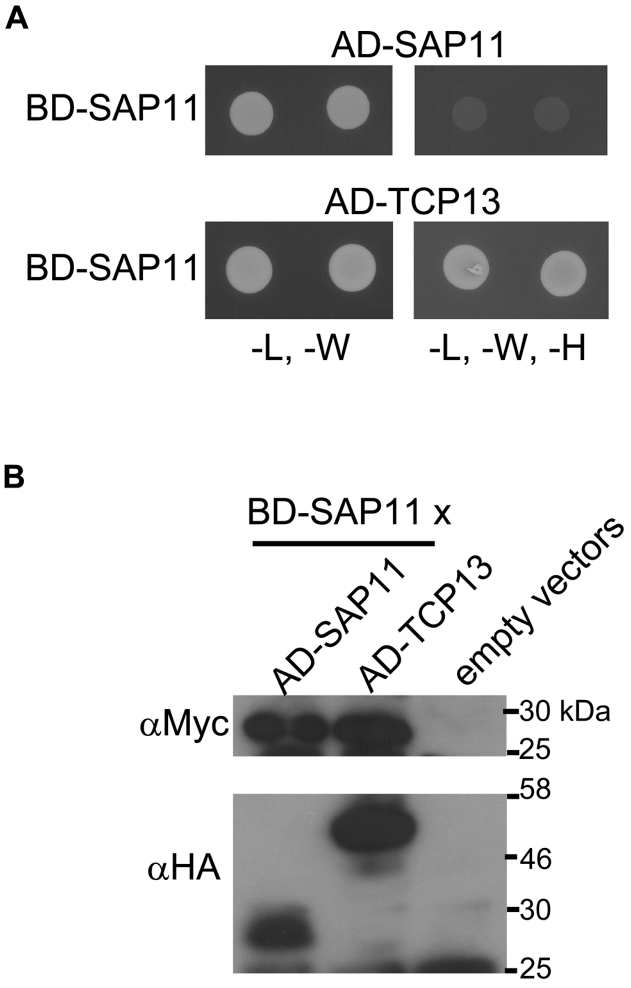

Figure S3. AY-WB phytoplasma SAP11 does not interact with itself. SAP11 was fused at the N-terminus to the DNA binding domain (BD) of GAL4 transcriptional activator (bait) and at the N-terminus to the transcription activation domain (AD) of GAL4 (prey). TCP13 fused to AD served as a positive control. Two independent yeast colonies were grown in Synthetic dropout (SD) media lacking leucine and tryptophan (-L , -W) or leucine, tryptophan and histidine (-L, -W, -H); the latter indicating interactions of bait and prey. **B**. Western blot hybridizations showing presence of bait and prey in yeast colonies of A. BD-SAP11 derivatives were detected by αMyc IgG and AD-SAP11 and AD-TCP13 fusion proteins with αHA IgG. Molecular weight markers (kDa) are indicated to the right of the blots.

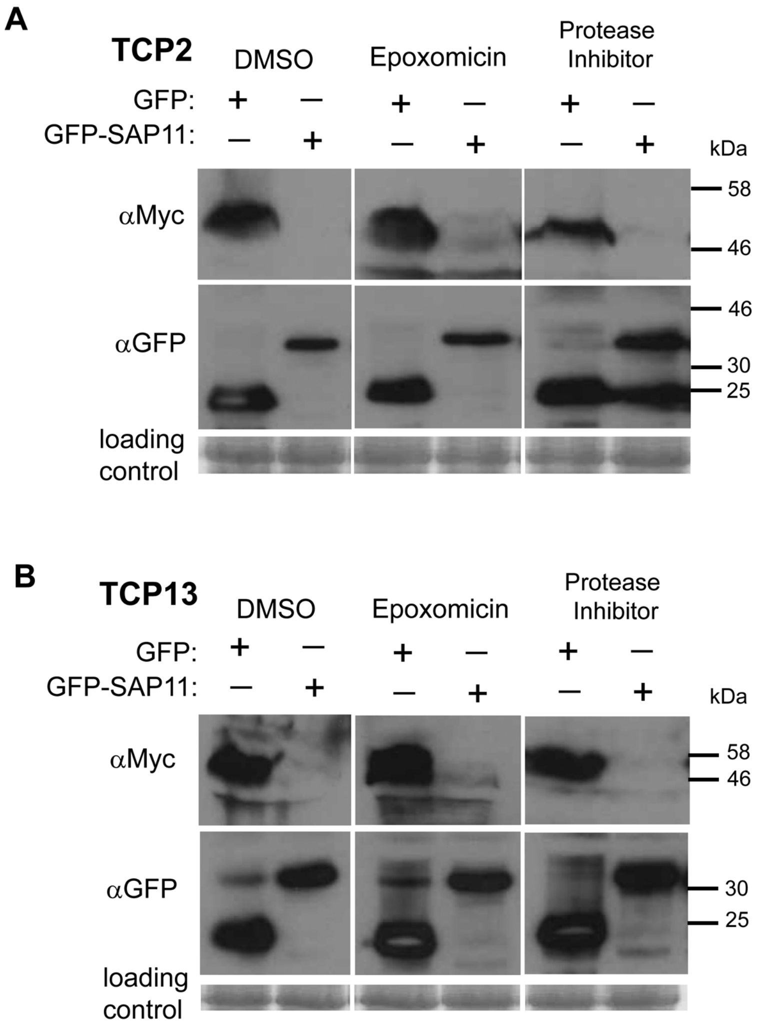

Figure S4. AY-WB phytoplasma SAP11-mediated destabilization of Arabidopsis TCP2 and TCP13 is not inhibited by protease or proteasomal inhibitors in *N. benthamiana* leaves. Co-expression of GFP, GFP-SAP11, and Myc-tagged TCP2 (A) and Myc-tagged TCP13 (B) in *N. benthamiana* leaves treated with 40 µM epoxomicin, protease inhibitor cocktail, or DMSO (mock control), as indicated. Samples were harvested 8 hours following treatment with the inhibitors or DMSO. GFP or GFP-SAP11 were detected with αGFP IgG and Myc-TCP2 or Myc-TCP13 were detected with αMyc IgG. The experiments were repeated twice with similar results. The loading control is Coomassie-stained Ribulose-1,5-bisphosphate carboxylase oxygenase large subunit. Molecular weight markers (kDa) are indicated to the right of the blots.

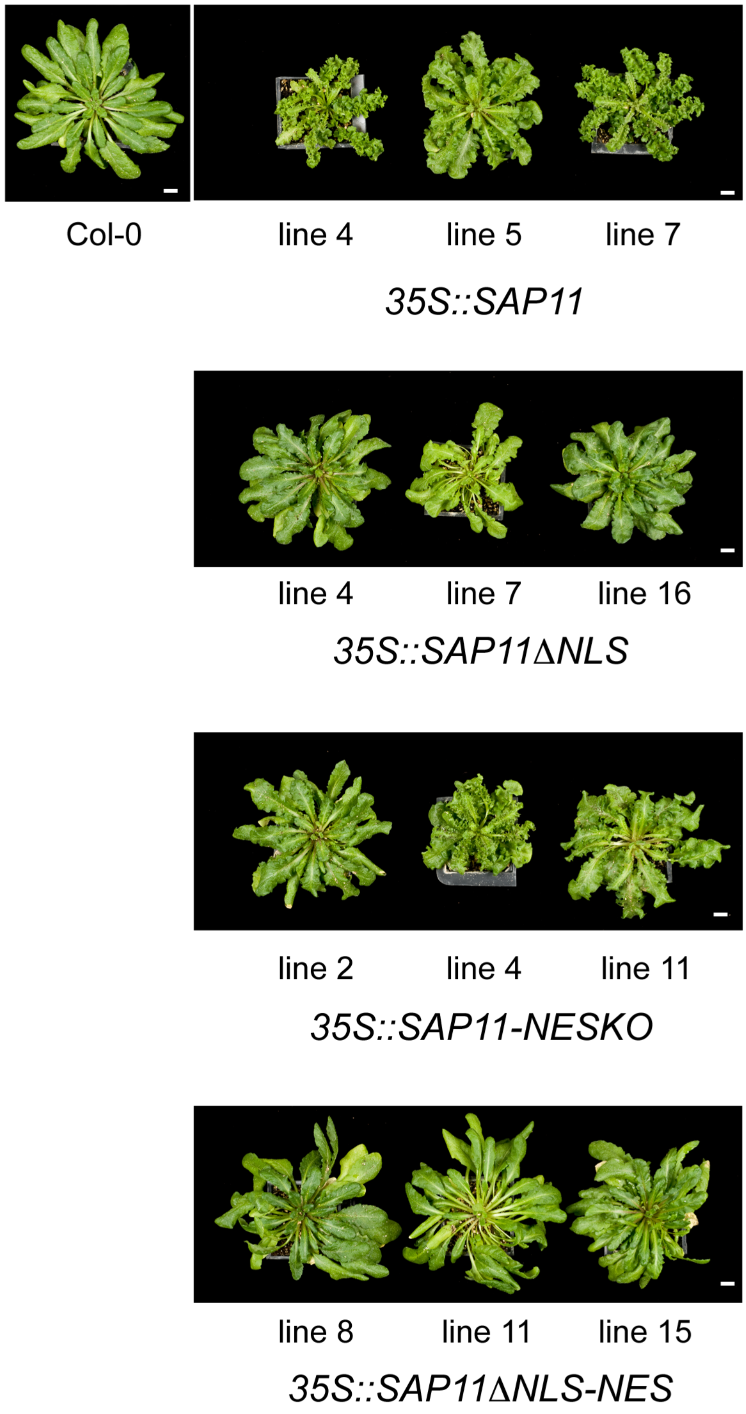

Figure S5. Arabidopsis transgenic lines expressing AY-WB phytoplasma SAP11 and SAP11 mutants. Photos of rosettes of Arabidopsis T3 transgenic lines at 6 weeks old grown at short day. Bars=1cm. Line numbers correspond to the sample numbers in **Figure 8**.

A

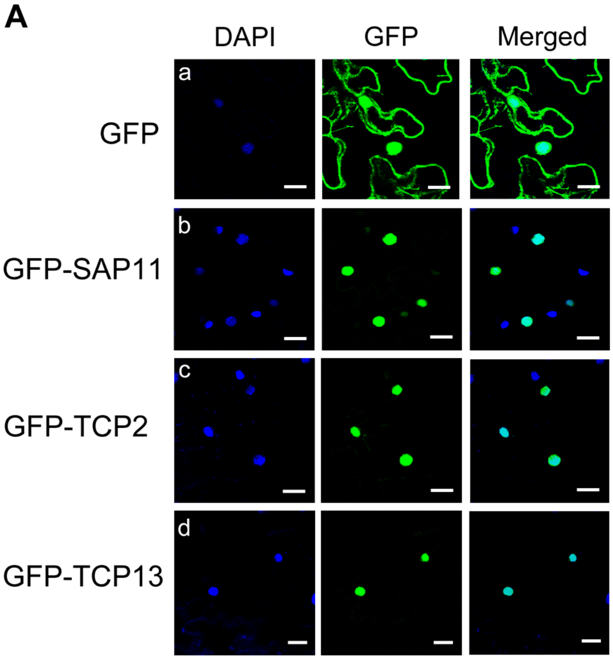

B

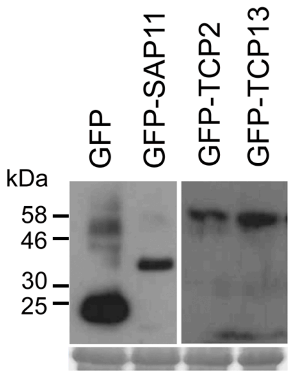

Figure S6. Arabidopsis TCP2 and TCP13 and AY-WB phytoplasma SAP11 are localized to the plant cell nuclei in *N. benthamiana* leaves. A. Confocal microscopy images showing subcellular distribution of (a) GFP; (b) GFP-SAP11; (c) GFP-TCP2; (d) GFP-TCP13. *N. benthamiana* leaves were agroinfiltrated with appropriate constructs and samples were examined under the confocal microscope after four days. Samples were stained with DAPI to indicate the positions of plant cell nuclei. Merged images comprise of overlays of GFP and DAPI fluorescence. Bars = 20 µm. B. Western blot hybridizations with αGFP IgG indicate the presence of full-length GFP-tagged proteins, with samples for microscopic and western blot analysis harvested from the same plants. Molecular weight markers (kDa) are indicated to the left of the blots.
